# Experimental Infection of Human Volunteers with the Heat-Stable Enterotoxin-Producing Enterotoxigenic *Escherichia coli* Strain TW11681

**DOI:** 10.3390/pathogens8020084

**Published:** 2019-06-22

**Authors:** Sunniva Todnem Sakkestad, Hans Steinsland, Steinar Skrede, Elisabeth Kleppa, Kristine Lillebø, Marianne Sævik, Hanne Søyland, Astrid Rykkje Heien, Marit Gjerde Tellevik, Eileen M. Barry, Halvor Sommerfelt, Kurt Hanevik

**Affiliations:** 1 Department of Clinical Science, Faculty of Medicine, University of Bergen, Postbox 7804, 5020 Bergen, Norway; sunniva.sakkestad@gmail.com (S.T.S.); steinar.skrede@helse-bergen-no (S.S.); 2 Centre for International Health, Department of Global Public Health and Primary Care, Faculty of Medicine, University of Bergen, Postbox 7894, 5020 Bergen, Norway; hans.steinsland@uib.no; 3 Department of Biomedicine, Faculty of Medicine, University of Bergen, Postbox 7804, 5020 Bergen, Norway; 4 Division for Infectious Diseases, Department of Medicine, Haukeland University Hospital, 5021 Bergen, Norway; kristine.lillebo@helse-bergen.no (K.L.); marianne.saevik@helse-bergen.no (M.S.); hanne.soyland@helse-bergen.no (H.S.); arheien@hotmail.com(A.R.H.); 5 Department of Microbiology, Haukeland University Hospital, 5021 Bergen, Norway; elisabethkleppa@gmail.com; 6 Norwegian National Advisory Unit on Tropical Infectious Diseases, Department of Medicine, Haukeland University Hospital, 5021 Bergen, Norway; marit.gjerde.tellevik@helse-bergen.no; 7 Center for Vaccine Development and Global Health, University of Maryland School of Medicine, Baltimore, MD 21201, USA; embarry@medicine.umaryland.edu; 8 Centre for Intervention Science in Material and Child Health (CISMAC), Department of Global Public Health and Primary Care, Faculty of Medicine, University of Bergen, Postbox 7804, 5020 Bergen, Norway; Halvor.Sommerfelt@uib.no; 9 Norwegian Institute of Public Health, 222 Oslo Postbox, Norway

**Keywords:** enterotoxigenic *Escherichia coli*, diarrhoea, controlled human challenge model, experimental infection, heat-stable enterotoxin, Colonization Factor Antigen I, YghJ

## Abstract

Infection with enterotoxigenic *Escherichia coli* (ETEC) producing the heat-stable enterotoxin (ST) is one of the most important causes of childhood diarrhoea in low- and middle-income countries. Here, we undertook a controlled human infection model (CHIM) study to investigate whether ST-producing ETEC strain TW11681 would be suitable for testing the protective efficacy of new ST-based vaccine candidates in vaccine challenge models. In groups of three, nine volunteers ingested 1 × 10^6^, 1 × 10^7^, or 1 × 10^8^ colony-forming units (CFU) of TW11681. Flow cytometry-based assays were used to measure CD4+ T cell responses and antibody levels targeting virulence factors expressed by the strain. We found that infection with TW11681 elicited few and mild symptoms, including mild diarrhoea in two volunteers, both of whom ingested 1 × 10^6^ CFU. Averaged across all volunteers, the CD4+ T cell responses specific for *E. coli* YghJ mucinase peaked 10 days after infection (3.2-fold (p = 0.016)), while the CD4+ T cell responses specific for Colonization Factor Antigen I (CFA/I) major fimbrial subunit (CfaB) peaked after 28 days (3.6-fold (p = 0.063)). The serum CfaB-specific anti-IgA and anti-IgG/IgM levels were significantly increased and peaked 3 months after infection. Both remained elevated for the duration of the 12-month follow-up. The corresponding anti-YghJ serological response was strongest after 10 days, although a significant increase was seen only for IgA levels (3.2-fold (p = 0.008)). In conclusion, due to its low diarrhoea attack risk, TW11681 is probably not suitable for testing the efficacy of new vaccines in human challenge studies at doses 1 × 10^6^ to 1 × 10^8^. However, the strain may still be useful in CHIMs for studying ETEC host-pathogen interactions.

## 1. Introduction

Infection with enterotoxigenic *Escherichia coli* (ETEC) is a major cause of diarrhoeal disease in low- and middle-income countries (LMICs) [1]. Developing an effective vaccine against ETEC has been a long-standing goal, and several candidates are in the development pipeline [1]. ETEC are defined as *E. coli* that produce one or both of the two protein enterotoxins called heat-stable toxin (ST), which is small and non-immunogenic, and heat-labile toxin (LT), which is large and immunogenic. Infections with ST-producing ETEC strains (with or without LT) are among the most important bacterial causes of moderate-to-severe diarrhoea in children under 5 years of age in LMICs. ST-ETEC is also associated with an increased risk of death [2]. Developing an effective ST-based vaccine, therefore, represents a promising strategy to confer broad protection against ETEC-induced diarrhoea. Two variants of ST exist, called porcine ST (STp) and human ST (STh). STh-producing ETEC are more pathogenic for young children in LMICs than STp-producing ETEC [3,4]. 

To estimate the efficacy of ST-based ETEC vaccine candidates in a controlled human infection model (CHIM) it is important that the challenge strain produces ST, but not LT, since the secretion of LT could mask an otherwise protective immune response to ST. It is also important that the strain reliably induces diarrhoea in most of the challenged individuals in order to minimise the number of volunteers needed to test the vaccines. In the close to five decades that have passed since the development of the first CHIM for ETEC, only one ST-only ETEC strain has been tested [5,6,7]. These trials were based on ETEC strain 214-4, which was isolated in 1975 from a 29 year-old traveller in Mexico who developed watery diarrhoea [8]. The strain induced diarrhoea in adult volunteers with attack risks of 60–80% when inoculum doses of 1 × 10^8^ and 1 × 10^10^ colony-forming units (CFU) were used [5,6]. It could, therefore, be suitable for use in a vaccine challenge model to test ST-based vaccines. However, since 214-4 has not been well characterized, and also is an STp-producer (Myron M. Levine, personal communication), it may not be as relevant for testing ST-based vaccines that target young children in LMICs, in whom STh-producing ETEC are epidemiologically much more important [3,4,9]. 

Here, we evaluate whether TW11681, which produces STh and represents an ETEC lineage commonly found associated with childhood diarrhoea, would be suitable for use in a vaccine challenge model. We experimentally infected 9 volunteers with TW11681 and followed them with daily clinical assessments as well as regular serum sample collection to evaluate their serum antibody responses to the ETEC Colonization Factor Antigen I (CFA/I) and to YghJ. YghJ is an *E. coli* mucinase and has in recent years been described as an important contributor to ETEC pathogenesis due to its ability to degrade the protective mucin layer covering the intestinal wall, allowing bacteria to access the epithelial cells for colonisation and endotoxin delivery [10]. Furthermore, YghJ was secreted by 89% of the isolates in a large collection of epidemiologically relevant ETEC strains [11], making it an attractive target for developing broadly protective vaccines against ETEC. 

## 2. Material and Methods

### 2.1. Volunteer Inclusion and Screening

We recruited 9 healthy young adults among students at the University of Bergen in Norway. Written information was distributed at the campus followed by oral presentations given in plenary sessions. We provided interested students with detailed oral and written information about the study. The inclusion criteria were age between 18 and 40, no travels to LMICs during the previous 12 months, passing a general health screening, no enteropathogens found in their stools, having normal baseline blood values, use of effective contraception for the female volunteers during the past 6 months, and being able to live in isolation for up to 10 days. The exclusion criteria were fever during the last 48 h before ingesting the dose, participation in other clinical trials during the last three months, use of immunosuppressive medicines, pregnancy, breastfeeding, positive fecal occult blood test, or a history of any chronic gastrointestinal condition. 

All volunteers completed a medical screening 20 to 48 (mean 32) days before ingesting the dose. The screening comprised a physical examination, an electrocardiogram recording, and collection of blood samples for analysis of full blood count, and electrolyte, creatinine, alanine aminotransferase, CRP, glucose, IgG, and IgA levels, as well as serum to exclude presence of hepatitis B virus antigen and antibodies against hepatitis C virus and HIV-1. We also collected stool samples for occult blood testing using Hemo-Fec® reagents (Med-Kjemi AS, Asker, Norway) and to screen for pathogenic bacteria and parasites. Female volunteers took a pregnancy test. A full medical history was taken, and the volunteers completed a questionnaire testing their comprehension of the study rationale, objectives, and procedures to be performed, as well as the possible risks and benefits. If all was acceptable, the volunteer signed the informed consent document. 

### 2.2. Setting

The volunteer study took place in October and November 2016 at the Infectious Diseases (ID) ward at the Division for Infectious Diseases at Haukeland University Hospital, where a system for experimentally infecting and housing three volunteers at a time had already been established [12]. The hospital guidelines for hygiene and safety, including enteric precaution guidelines, were carefully followed by the volunteers and the study personnel. 

### 2.3. Strain Description

The process of selecting a strain for use in the model was based on evaluation of phylogenetic data [13] and subsequent whole genome sequencing and analysis of relevant strains [14] (GenBank BioProject: PRJNA59749). We chose ETEC strain TW11681 because it belongs to an epidemiologically relevant ETEC lineage (ETEC8 [13], L6 [15]) that is often found associated with childhood diarrhoea in LMICs and because it has a serotype (O19:H45) and encoded ETEC virulence factors (STh, CFA/I, and Coli Surface antigen 21 [CS21]) that are common to strains from this lineage. The strain was originally isolated in Guinea-Bissau in 1997 from the stool of a 6-month-old girl who had diarrhoea [4], and has since been stored at −70 °C. It is sensitive to ciprofloxacin and trimethoprim-sulfamethoxazole.

### 2.4. Preparation of Inocula

From the single-colony pick culture used for the whole-genome sequencing of TW11681, the Preparation Laboratory of the Center for Vaccine Development at the University of Maryland School of Medicine prepared the master cell bank and subsequent working cell bank (WCB) that was used for preparing the inoculum as previously described [12]. Briefly, TW11681 from a WCB tube was streaked onto animal product-free Luria-Bertani (LB) agar, and six colonies from these plates were picked, mixed together in phosphate buffered saline (PBS), and pipetted onto thick LB agar dishes to produce a lawn. Two hours before the volunteers were to ingest the dose, the bacterial culture was harvested and washed three times in PBS before the cell concentration was measured by absorbance spectrometry at 600 nm. The cells were then diluted in PBS to the required dose concentration, and 2 mL doses were transferred to individual 50 mL Falcon tubes to be used in the experimental infection. Dose verification was done by serial dilution of the dose in PBS and plating in triplicate onto LB agar, followed by back-calculation of the TW11681 concentration from the resulting colony counts. 

### 2.5. Experimental Infection

After an overnight fast, the volunteers were admitted to the ID ward on the day of ingesting the dose. To neutralize gastric acid, the volunteers drank 120 mL 1.33% bicarbonate buffer one minute before ingesting the dose. Thirty mL bicarbonate buffer was added to the 2 mL dose immediately before drinking, and the volunteers were allowed to eat and drink normally 90 min afterwards. The dose was increased 10-fold for each new group of three volunteers, starting at 1 × 10^6^ CFU for the first group. This relatively low starting dose was chosen due to safety concerns. TW11681 has never before been administered to volunteers, and the gradual group-wise dose escalation allowed for a controlled evaluation of symptoms before deciding on whether a dose increase was justifiable. Based on results from previous studies and experiences, experimental ETEC infection may well induce diarrhoea in volunteers with doses as low as 10^6^ CFU [12,16]. To clear the infection, the volunteers were treated with 500 mg ciprofloxacin two times daily for three days, starting on day 5, but earlier treatment would be given if the volunteers experienced severe or moderate diarrhoea lasting for ≥24 h, or had mild diarrhoea and two or more of the following symptoms for two days: fever, vomiting, abdominal pain or cramping, headache, myalgias, or nausea. The volunteers continued to be hospitalized and adhered to enteric precaution guidelines as long as ETEC was still detected by real-time PCR in daily collected stool specimens, as described below. When three consecutive stool specimens were ETEC negative, the volunteer was discharged from the ID ward and further follow-up was performed in the outpatient clinic. 

### 2.6. Clinical Evaluation 

Dedicated study nurses and other clinical staff visited the volunteers at least three times daily to record their vital signs including body temperature, blood pressure, and heart rate, as well as to assess their general health and wellbeing. Axillary temperature was measured by using a Bosotherm Basic thermometer (Bosch + Sohn GmbH und Co., Jungingen, Germany), and readings of ≥38 °C was considered to represent fever. The volunteers documented symptoms using self-report forms, which were reviewed together with the study physician on a daily basis. Symptoms specifically asked for were nausea, abdominal pain, abdominal cramping, flatulence, bloating, vomiting, constipation, decreased appetite, headache, malaise, fever, chills, myalgias, and lightheadedness. Signs of hypovolemia were recorded, as well as any other organ- or body system-related symptoms. The severity of each symptom was graded as follows: Grade 1 (mild; no disruption of normal daily activities; relieved with symptomatic treatment), grade 2 (moderate; affected or reduced daily activity; only partially relieved by symptomatic treatment), and grade 3 (severe; considerably affected or reduced daily activity; not relieved by symptomatic treatment). 

All stools were collected and weighed in a plastic single-use receptacle, and stool fluidity was graded as follows: Grade 1 (firm, formed), grade 2 (soft, formed), grade 3 (viscous opaque liquid or semiliquid which assumes the shape of the container), grade 4 (watery, non-viscous, opaque liquid which assumes the shape of the container) and grade 5 (clear or translucent, watery or mucoid liquid which assumes the shape of the container). A volunteer was defined as having diarrhoea when he or she produced 1 loose/liquid stool (grade ≥3) totalling ≥300 g, or ≥2 loose/liquid stools totalling ≥200 g during any 48-h period within 120 h after challenge. A diarrhoeal episode was graded as mild if the subject produced 1–3 loose stools totalling 200–400 g, moderate with 4–5 loose stools or a total weight of 401–800 g, and severe with ≥6 loose stools or a total weight of >800 g [17]. Diarrhoea attack risk was calculated by dividing the number of volunteers who developed diarrhoea by the total number of volunteers in the given group.

To assess the overall severity of the disease induced by the infection, we scored subjective symptoms, objective signs, and diarrhoeal output and added the outcomes to get the disease severity score, according to Porter et al. [18]. The score component outcomes were as follows: Objective signs (0 = No vomiting AND no fever, 1 = 1 episode of vomiting AND no fever, 2 = >1 episode of vomiting/24 h OR any fever); Subjective symptoms (0 = No subjective symptoms, 1 = Mild lightheadedness OR mild-moderate nausea, malaise, headache, or abdominal cramps, 2 = Moderate-severe lightheadedness OR severe nausea, malaise, headache, or abdominal cramps); Diarrhoea score in maximum 24 h period with loose/liquid stools (0 = No loose/liquid stools, 1 = >0 to ≤400 g OR 1 to 4 loose/liquid stools, 2 = >400 to ≤600 g OR >4 to ≤7 loose/liquid stools, 3 = >600 to ≤1,000 g OR >7 to 12 loose/liquid stools, 4 = >1000 g OR >12 loose/liquid stools).

### 2.7. Specimen Collection

Blood was collected by venepuncture on the day of screening or immediately before ingesting the dose, and at days 7, 10, and 28, as well as 3, 6, and 12 months afterwards. Peripheral blood mononuclear cells (PBMCs) were isolated from whole blood immediately before and 7 days after ingesting the dose by using mononuclear cell preparation tubes (BD Vacutainer CPT, BD Biosciences) and the concentration of PBMCs were determined by using a C6 Accuri flow cytometer (BD Biosciences) before lymphocyte supernatants (ALS) were prepared as previously described [19]. 

### 2.8. Detection of Enterotoxigenic Escherichia coli (ETEC) in Stool Specimens

Stool specimens or, if not available, rectal swabs were collected and analyzed for the presence of ETEC daily. The specimens were streaked onto lactose agar before incubation aerobically at 35 °C overnight in order to identify *E. coli*-like colonies. Using a 1 μL inoculation loop, a representative part of the bacterial colonies was collected and suspended in 0.5 mL of distilled water. The suspension was first boiled for 10 min and then centrifuged 13,000× g for 5 min. We added 5 μL of the supernatant to 15 μL of reaction mixture (LightCycler FastStart DNA Master^PLUS^ SybrGreen (Roche Diagnostics GmbH, Germany)) containing 0.5 μM of each primer. The ST-gene was detected by real-time-PCR as earlier described [12]. The primers used were the ST-gene_forward_ (JW7): 5’-CAC-CCG-GTA-CAR-GCA-GGA-TT-3’, and the ST-gene_reverse_ (JW14) 5’- ATT-TTT-MTT-TCT-GTA-TTR-TCT-T-3’ [20]. The PCR was run on a LightCycler 2.0 (Roche Diagnostics) instrument. A pre-heating step at 95 °C for 10 min was followed by 40 cycles of 10 seconds at each of the temperatures 95, 55, and 72 °C for denaturation, annealing, and extension, respectively. Finally, a melting curve analysis at 60 to 95 °C with a temperature transition rate of 0.1 °C/second was done to determine the melting temperatures for the PCR products. A positive control consisting of extracted DNA from the sample strain, and a negative control containing distilled water, were used in each run. Each melting curve was evaluated and compared with the positive and negative controls, and a specimen was considered positive if the ST-gene was detected. 

### 2.9. Antigen Preparations

We amplified and ligated the structural genes for CfaB (UniProtKB ID: E3PPC4) and YghJ (UniProtKB ID: P0CK95) from TW11681 into pET-30 (Thermo Fisher Scientific Inc. Waltham, MA). CfaB is the main structural subunit of Colonization Factor Antigen I (CFA/I), which is a fimbrial colonisation factor that helps anchor ETEC to the host intestine, while YghJ is a secreted mucinase that most pathogenic *E. coli* are capable of producing and that helps break down the protective mucus barrier in the small intestines [10]. To produce the proteins, we used ClearColi BL21(DE3) (Lucigen Corporation, Middleton, WI) to avoid co-purifying *E. coli* lipopolysaccharides that could also stimulate T cells. The proteins were purified from cleared cell lysate by His-Tag purification, followed by dialysis against phosphate buffered saline, pH 7.4. Protein concentration was estimated by using the Micro BCA Protein Assay Kit (Thermo Fisher Scientific), and purity and protein size was assessed by SDS-PAGE. YghJ is around 166 kDa, while CfaB is around 26 kDa.

### 2.10. T Cell Assay and Flow Cytometry

To evaluate specific CD4+ T cell responses to the infection, we cultured whole blood from before and 10 days, 28 days, and 3 months after ingesting the dose in the presence of CfaB and YghJ antigens and measured the percentage of CD4+ T cells that co-expressed CD134 (OX40) and CD25 by flow cytometry [21,22]. The method is considered to be a robust technique to identify antigen-specific CD4+ T cells, since it is only minimally affected by bystander activation [23]. In these assays, we diluted 10 µg each of purified CfaB and YghJ protein in 500 µL X-VIVO 15 Serum-free Hematopoietic Cell Medium with Gentamicin and Phenol Red (Lonza Ltd, Basel, Switzerland) and stored these at −80 °C in 24-well culture plates (Corning Incorporated, Corning, NY) until use. We used the cell medium without added antigen as a negative control, and cell medium with added 0.1 µg Staphylococcal Enterotoxin B (SEB) (Sigma-Aldrich, St. Louis, Missouri) as a positive control. After collecting blood from the volunteers, a cell-culture plate was thawed and 500 µL of Sodium Heparin anticoagulated whole blood from each volunteer was added to antigen and control wells followed by gently mixing. The plates were then incubated at 37 °C and 5% CO_2_ for 42–48 h. We then transferred 500 µL of the culture to separate tubes and lysed erythrocytes by adding a hypotonic buffer containing ammonium chloride, sodium bicarbonate, and EDTA, after which we centrifuged the plates at 350× g for 5 min before discarding the supernatants. The remaining pelleted cells were then washed once in ammonium chloride and once in PBS, each step followed by centrifugation at 300× g for 5 min and removal of the supernatant. 

The cells were stained in 96-well plates (Corning Incorporated, Corning, NY USA) by adding fluorochrome-conjugated monoclonal antibodies targeting CD3, CD4, CD8a, CD25, CD134, and CD14 (AF700-CD3, Clone HIT3a [Cat#300324, BioLegend, San Diego, CA]; BV510-CD4, Clone OKT4 [Cat#317444, BioLegend]; FITC-CD8a, Clone HIT8a [Cat#300906, BioLegend]; APC-CD25, Clone M-A251 [Cat#555434, BD BioSciences, San Jose, CA]; PE-CD134, Clone ACT35 [Cat#555838, BD BioSciences]; and Pe-Cy5-CD14, Clone 61D3 [Cat#15-0149-42, Thermo Fisher Scientific, Waltham, MA], respectively) in addition to the 7-AAD Viability Staining Solution (Cat#555816, BioLegend). After incubation for 15 min in the dark, the labelled cells were washed once in PBS, before being resuspended in 2% paraformaldehyde. The cells were counted on an LSR Fortessa flow cytometer (BD Biosciences). Forward and side scatter was used to identify singlet lymphocytes, and monocytes and dead cells were excluded by using the CD14 and 7-AAD signals. CD4+ T cells were identified by the expression of CD3 and CD4, and out of these we determined the percentage of CD25-CD134 doubly positive cells as a measure of activated antigen-specific CD4+ T cells. The data were analyzed by using FlowJo (FlowJo, LCC), and Wilcoxon matched-pairs signed rank test (GraphPad Prism, La Jolla, CA) was used to test for differences in the percentage of antigen-specific cells between different blood specimens. 

### 2.11. Antibody Assays

To investigate antibody responses to the challenge strain in serum and in ALS, we performed a multiplex bead immunoassay. Briefly, CfaB, YghJ, and glutathione S-transferase (GST) were covalently coupled to 5 µm Cyto-Plex carboxylated polystyrene particles (Thermo Fisher Scientific) of different fluorescence intensities. The GST was used as a negative control. We thawed serum specimens from all 9 volunteers collected on days 0, 10 and 28, as well as 3, 6 and 12 months after ingesting the dose, and diluted them 1:50 in Assay Buffer (PBS containing 1% BSA and 0.05% Tween-20). Likewise, ALS specimens collected on days 0 and 7 were thawed and diluted 1:4 in Assay Buffer. Around 6000 beads for each of the three proteins were added to wells of MultiScreen_HTS_ HV 0.45 µm filter plates (Merck) and incubated with serum or ALS samples at room temperature for one hour. The beads were then washed and incubated with the secondary antibody; IgG/M (Alexa Fluor 488-AffiniPure F(ab)2 Fragment Goat Anti-Human IgG + IgM [Jackson Immunoresearch, West Grove, PA]) or IgA (Alexa Fluor 488-AffiniPure Goat Anti-Human Serum IgA [Jackson Immunoresearch]). Following incubation, the beads were washed and finally analyzed on an LSR Fortessa flow cytometer. For each protein and control, we calculated the FITC median fluorescence intensity (MFI) value using FlowJo. The data were adjusted by subtracting the MFI value of GST from that of CfaB and YghJ. GraphPad Prism was used to analyze the data and calculate MFI fold changes between time points for the two antigens, and Wilcoxon matched-pairs signed rank test was used to test for differences in antibody levels between different timepoints. 

### 2.12. Ethics Approval

The study was approved by the Regional Committee for Medical and Health Research Ethics, Health Region West (REC-West) with protocol identification number 2014-826, and registered in ClinicalTrials.gov NCT02870751. The study was monitored by an independent monitor.

## 3. Results

### 3.1. Volunteer Characteristics 

Of the 9 volunteers who participated in the study, 8 were female. They had a mean age of 25.7 years (range: 23.8, 28.5), and a mean body mass index of 21.7 kg/m^2^ (range: 18.6, 25.1).

### 3.2. Clinical Results

The 9 volunteers were admitted to the ID ward in successive groups of three, and were given 1 × 10^6^ (first group), 1 × 10^7^ (second group) and 1 × 10^8^ (third group) CFU TW11681. ETEC was detected in the stools for 5–8 days (3 days for volunteer EV14) after they ingested the dose, suggesting all volunteers were successfully colonized by the challenge strain. Two of the 9 volunteers developed mild diarrhoea (both at doses 1 × 10^6^ CFU) after a mean incubation period of 58 h (range: 35, 80) (Table 1). Both diarrhoeal episodes were short and mild, and no volunteers reached the criteria for early antibiotic treatment. The most frequently reported symptoms were abdominal pain (five volunteers [56%]) and abdominal cramping (four volunteers [44%]) (Table 2). All the reported signs and symptoms were mild or moderate. The resulting disease severity scores were relatively low, with only EV10 having a higher score than 2 (Table 3). None of the volunteers became moderately or severely dehydrated and none of them needed oral rehydration salts solutions or transfusion of intravenous fluid. There were no serious adverse events. Since the diarrhoeal episodes observed were only mild, and none of the volunteers experimentally infected with the two highest doses developed diarrhoea, we decided to end further recruitment to the study and not increase the dose to 1 × 10^9^ CFU. 

### 3.3. CD4+ T Cell Responses

**Figure 1:**
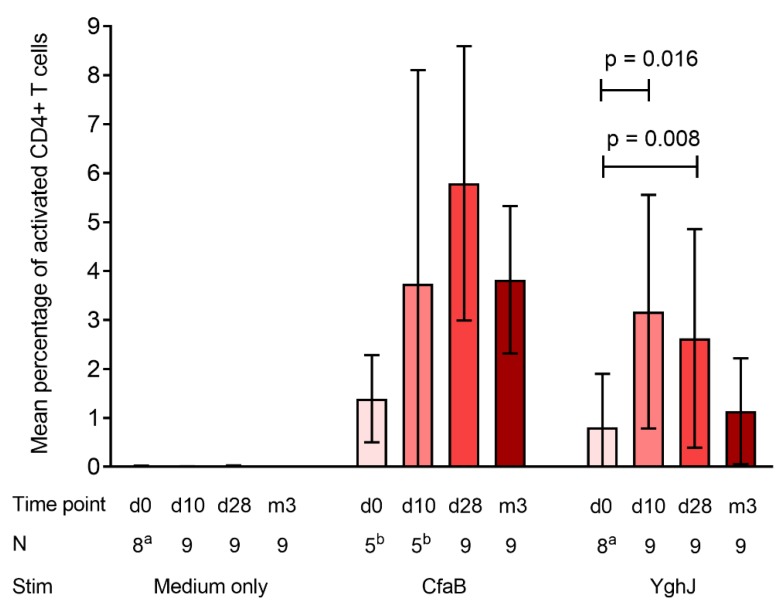
Antigen-specific CD4+ T cell responses in peripheral blood following *in-vitro* stimulation with antigens CfaB and YghJ. The responses were measured by flow cytometry as the mean percentage of CD4+ T cells expressing both CD25 and CD134, and are based on samples from 9 adult healthy volunteers at different time points before (day 0) and after (day 10, day 28, 3 months) ingesting the dose. The bars represent the mean percentage of CD4+ T cells across all volunteers after incubation with medium (negative control), CfaB, and YghJ, and the error bars represent the corresponding 95% confidence intervals of the means. The horizontal bars indicate significant increases in YghJ-specific CD4+ T cells from day 0 to day 10 and day 28, respectively. Abbreviations: N: Number of volunteers, Stim: Antigen used for stimulation. ^a^ Assay results from 1 volunteer was excluded due to high levels of non-specific background signals. ^b^ Assay was performed only on specimens from 5 volunteers due to limited access to purified CfaB.

We found a non-significant 2.7-fold increase (p = 0.313) in the mean percentage of CfaB-specific CD4+ T cells in peripheral blood from day 0 to day 10 after ingesting the dose, while the percentage increased slightly to 3.6-fold (p = 0.063) from day 0 to day 28 (Figure 1). It should be noted that CfaB-specific CD4+ T cell responses at day 0 and day 10 were based only on specimens from 5 volunteers, due to limited access to purified CfaB. Correspondingly for YghJ, we found a significant 3.2-fold increase (p = 0.016) from day 0 to day 10 and a 2.6-fold increase (p = 0.008) from day 0 to day 28. The percentage of YghJ-specific cells then returned to baseline levels while the CfaB-specific levels seemed to persist longer. The SEB positive control activated 28.9% of the CD4+ T cells, averaged across all volunteers and time points. 

### 3.4. Serum and Lymphocyte SupernatantAntibody Responses Against TW11681 Antigens 

**Figure 2:**
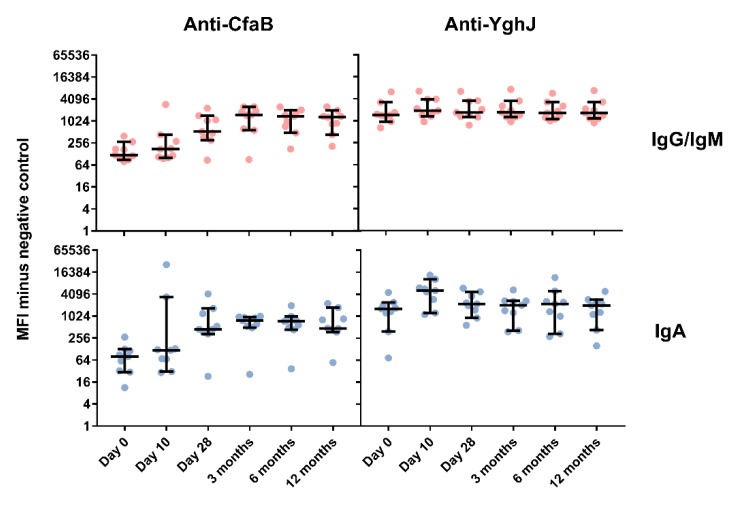
CfaB- and YghJ-specific IgG/IgM and IgA antibody levels in serum from nine healthy adult volunteers at different time points after ingesting enterotoxigenic *Escherichia coli* (ETEC) strain TW11681. The antibody levels were measured in a bead-based flow-cytometry assay, and are presented as the median fluorescence intensity (MFI) values minus the MFI of the negative controls. Circles represent the antibody levels of each volunteer; the middle horizontal bars represent the medians; the whiskers bridge 95% confidence intervals of the median.

In serum, 8 of the 9 volunteers (89%) exhibited an increase in antibody responses against CfaB compared to pre-infection levels, and the maximum MFI fold increase was seen 3 months after ingesting the dose, with a 9.6-fold increase in antigen specific IgA (p = 0.008) and a 12.5-fold increase in antigen specific IgG/IgM (p = 0.008) (Figure 2). The levels remained elevated until the end of the 12-month follow-up period, with 5.9-fold (p = 0.004) and 10.9-fold (p = 0.004) increases for IgA and IgG/IgM, respectively. The corresponding YghJ responses were less pronounced for IgG/IgM with only two volunteers developing a more than two-fold response compared to pre-infection levels. However, 7 of the 9 volunteers (78%) developed a more than 2-fold increase in anti-YghJ serum IgA levels. The two strongest responders were also the same two volunteers who developed an IgG/IgM response and who developed diarrhoea. The maximum increase in YghJ-specific antibody levels was seen from day 0 to day 10, with a significant 3.2-fold increase in IgA (p = 0.008) and a small and non-significant 1.3-fold increase in IgG/IgM (p = 0.074). In general, the volunteers had relatively high levels of YghJ-specific antibodies even before being infected, although it is possible that some of this signal could be non-specific.

**Figure 3:**
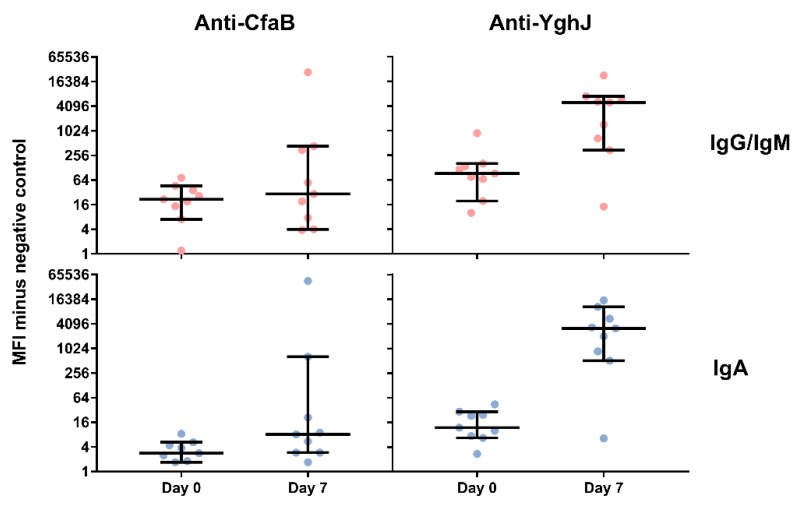
CfaB- and YghJ-specific IgG/IgM and IgA antibody levels in lymphocyte supernatants (ALS) from nine healthy adult volunteers at different time points after ingesting enterotoxigenic *Escherichia coli* (ETEC) strain TW11681. The antibody levels were measured in a bead-based flow-cytometry assay, and are presented as the median fluorescence intensity (MFI) values minus the MFI of the negative controls. Circles represent the antibody levels of each volunteer; the middle horizontal bars represent the medians; the whiskers bridge 95% confidence intervals of the median.

In ALS, there was a pronounced increase in antibody levels against YghJ from day 0 to day 7, with a 267-fold increase for IgA (p = 0.004) and a 55-fold increase for IgG/IgM (p = 0.004) averaged across all volunteers. The corresponding CfaB responses were relatively weak for most of the volunteers with a 2.9-fold increase for IgA (p = 0.04) and a moderate and uncertain increase of 1.3-fold for IgG (p = 0.250). However, two volunteers, both of whom had mild subjective symptoms to the experimental infection, but no development of diarrhoea, developed consistently strong CfaB-specific IgG/IgM and IgA responses (Figure 3).

## 4. Discussion

In this study we evaluated whether the ST-only ETEC strain TW11681 could be suitable for use in a vaccine challenge model. We found that the volunteers were colonized with the strain and developed immune responses against the CfaB and YghJ virulence proteins that the strain produces, including specific CD4+ T cell responses as well as serum and ALS IgG/IgM and IgA antibody responses. However, only two of the nine volunteers (22%) developed diarrhoea, and the risk of developing diarrhoea did not increase with increasing dose up to 1 × 10^8^. In general, aiming for strains and doses that give attack risks of 70–75% is regarded as favorable to achieve statistical significance in small volunteer vaccine challenge models, while at the same time not overwhelming otherwise protective immune responses in vaccinated volunteers [17,24]. In contrast to the apparent low risk of diarrhoea, abdominal pain and cramping were frequently reported (56 and 44%, respectively), and these symptoms were experienced by a higher proportion of the volunteers than is normally observed in CHIM experiments with other ETEC strains at these relatively low doses [16]. Since the two volunteers who developed diarrhoea both received 1 × 10^6^ CFU, and since none of the volunteers developed loose stools at either 1 × 10^7^ or 1 × 10^8^ CFU, it seems unlikely that further increasing the dose would dramatically increase the diarrhoea attack risk for this strain. For these reasons, we believe TW11681 will not be suitable for use in vaccine challenge models. 

In a separate study, the concentrations of TW11681 DNA in daily collected stool specimens from these volunteers were determined using a qPCR assay that was specific for TW11681 [25]. Here it was shown that six of the volunteers, including the two who developed diarrhoea, appeared to have substantial increases in stool TW11681 DNA concentrations in the days following dose ingestion. The two volunteers who developed diarrhoea did not have higher stool TW11681 DNA concentrations than the other four volunteers, suggesting a lack of diarrhoea may not necessarily be a result of suboptimal colonisation. Further studies are needed to identify the underlying reasons for why this strain often failed to induce diarrhoea even when the degree of colonisation seemed to be high. The three volunteers who did not have a substantial increase in stool TW11681 DNA concentrations tended to have weak serum antibody responses to CFA/I and YghJ, and this may help to explain some of the variation in the immune responses seen in the present study. 

The three strains most commonly used in ETEC CHIMs, H10407, E24377A, and B7A originate from different ETEC lineages and exhibit high diarrhoea attack risks following ingestion of relatively low CFU doses [16]. However, they express both LT and ST and are therefore not suitable for use in evaluating the protective efficacy of ST-based vaccine candidates. In previous CHIM studies with the ST-only ETEC strain 214-4, attack risks of 0% (1 × 10^6^ CFU), 60-80% (1 × 10^8^ CFU) and 80% (1 × 10^10^ CFU) were reported [6,7]. Although 214-4 is an STp-producer, these studies show it is possible to obtain a high diarrhoea attack risk with an ST-only strain. Therefore, we plan to identify a new representative STh-only ETEC strain from a different lineage and test it in another volunteer challenge study.

Even though only two of the volunteers developed diarrhoea, all nine were successfully colonized by the challenge strain, and most of them mounted strong immune responses to the CfaB and YghJ virulence factors of the challenge strain. YghJ responses were faster to develop, but they were also more short-lived. This may be explained by YghJ also being expressed by other strains of *E. coli* [10] and it is therefore likely that volunteers were pre-exposed to this antigen. Successful intestinal colonization by TW11681 [25], and thus increased antigen exposure, seems the most likely explanation for strong YghJ responses since also volunteers with no diarrhoea (but with a high degree of colonization) developed good anti-YghJ IgA responses. 

The time it took to mount a strong anti-CfaB antibody response in ALS and in serum varied between volunteers, with only six volunteers showing a strong serum response 28 days after ingesting the dose. This suggests that most of these volunteers may not have experienced prior infections with CFA/I-producing ETEC strains or that they have little immunological memory of such infections. As expected, the CfaB-specific cell-mediated response was faster, with a substantial increase in CfaB-specific CD4+ T cells on day 10. Unlike the estimates for YghJ, this CD4+ T cell increase for CfaB did not reach statistical significance, which is probably due to the low number of observations and the large variability seen for CfaB in the T cell assay. Globally, CFA/I is one of the most common colonisation factors expressed by ETEC, and estimates show that it is isolated from 20% of the non-travel population with ETEC diarrhoea, thus being an attractive target for ETEC vaccine development [26]. It is therefore encouraging to observe long-lasting CFA/I specific cellular and humoral immune responses from an experimental ETEC infection.

## 5. Conclusions 

Although ETEC strain TW11681 may not be suitable for use in vaccine challenge models due to low attack risk of diarrhoea, the clinical results obtained from this highly controlled study, complemented by a thorough and long-term follow-up of immunological responses, provide useful new insights into the pathogenicity of ST-only ETEC strains. The strain elicited non-diarrhoeal abdominal symptoms, successfully colonized the volunteers and induced relatively strong, yet varied antibody and T cell responses, suggesting it could be useful in CHIMs to study ETEC host-pathogen interactions. The findings presented in this study thus provide a useful reference for the continued work to develop models for testing the protective efficacy of forthcoming ST-only toxoid-based vaccine candidates.

## Figures and Tables

**Table 1:** Description of diarrhoeal episodes among 9 volunteers experimentally infected with enterotoxigenic *Escherichia coli* (ETEC) strain TW11681.

Target Dose (CFU)	No. of Volunteers	No. with Diarrhoea	Attack Risk	Median Severity	Mean Incubation Period, Hours (Range)	Mean 24h Maximum Stool Output, Grams (Range)	Mean Whole Episode Stool Output, Grams (Range)	Mean Episode Duration, Hours (Range)	Mean 24h Maximum Stool Output, Count (Range)
1 × 10^6^	3	2	67%	Mild	58 (35–80)	364 (289–389)	364 (289–389)	1.3 (0–2.5)	1.5 (1–2)
1 × 10^7^	3	0	0%	NA	NA	NA	NA	NA	NA
1 × 10^8^	3	0	0%	NA	NA	NA	NA	NA	NA

**Table 2:** Symptoms and signs in 9 volunteers experimentally infected with enterotoxigenic *Escherichia coli* (ETEC) strain TW11681.

Symptom	Dose (CFU)			Combined (% of Volunteers)
	1 × 10^6^	1 × 10^7^	1 × 10^8^	
Diarrhoea	2	0	0	2 (22%)
Nausea	1	0	0	1 (11%)
Abdominal pain	2	1	2	5 (56%)
Abdominal cramping	2	0	2	4 (44%)
Flatulence	1	1	1	3 (33%)
Decreased appetite	1	1	0	2 (22%)
Bloating	1	0	1	2 (22%)
Headache	1	0	0	1 (11%)
Malaise	1	1	0	2 (22%)
Lightheadedness	1	0	0	1 (11%)

None of the volunteers experienced vomiting, constipation, fever, chills, myalgias, or hypovolemia.

**Table 3:** Disease severity score among 9 volunteers experimentally infected with enterotoxigenic *Escherichia coli* (ETEC) strain TW11681.

Dose (CFU)	Volunteer ID	Score Components			Disease Severity Score (0–8)
		Objective Signs (0–2)	Subjective Symptoms (0–2)	Diarrhoea Score (0–4)	
1 × 10^6^	EV10	0	2	1	3
1 × 10^6^	EV11	0	1	1	2
1 × 10^6^	EV12	0	0	0	0
1 × 10^7^	EV13	0	0	0	0
1 × 10^7^	EV14	0	0	0	0
1 × 10^7^	EV15	0	1	0	1
1 × 10^8^	EV16	0	1	0	1
1 × 10^8^	EV17	0	0	0	0
1 × 10^8^	EV18	0	1	0	1
